# Heterologous overexpression of *Apocynum venetum* flavonoids synthetase genes improves *Arabidopsis thaliana* salt tolerance by activating the IAA and JA biosynthesis pathways

**DOI:** 10.3389/fpls.2023.1123856

**Published:** 2023-03-27

**Authors:** Mengchao Zhang, Xueli Lu, Tingting Ren, Prince Marowa, Chen Meng, Juying Wang, Hui Yang, Chunhua Li, Li Zhang, Zongchang Xu

**Affiliations:** ^1^ College of Agriculture, Shanxi Agricultural University, Taigu, China; ^2^ Marine Agriculture Research Center, Tobacco Research Institute of Chinese Academy of Agricultural Sciences, Qingdao, China; ^3^ Department of Plant Production Sciences and Technologies, University of Zimbabwe, Harare, Zimbabwe; ^4^ Service Center for Comprehensive Utilization of Saline-Alkali Land in Agricultural High-tech Industrial Demonstration Zone of the Yellow River Delta, Dongying, China; ^5^ Industry Promotion Service Center of Agricultural High-tech Industrial Demonstration Zone in the Yellow River Delta, Dongying, China

**Keywords:** *apocynum venetum*, salt stress, ROS, flavonoids, JA, IAA

## Abstract

Salt stress is a serious abiotic stress that primarily inhibits plant growth, resulting in severe yield losses. Our previous research found that flavonoids play important roles in *A. venetum* salt stress tolerance. In response to salt stress, we noted that the flavonoid content was depleted in *A. venetum*. However, the detailed mechanism is still not clear. In this study, the expression patterns of three flavonoids synthetase genes, *AvF3H*, *AvF3’H*, and *AvFLS* were systemically analyzed under salt stress in *A. venetum* seedlings. The salt tolerance of transgenic *Arabidopsis* plants was improved by heterologous overexpression of these synthetase genes. The NBT and DAB staining results as well as H_2_O_2_ and O_2_•^-^ content analysis revealed that under salt stress, ROS molecules were reduced in transgenic plants compared to WT plants, which corresponded to the activation of the antioxidant enzyme system and an increase in total flavonoid content, particularly rutin, eriodictyol, and naringerin in transgenic plants. External application of flavonoids reduced ROS damage in WT plants just like what we observed in the transgenic plants (without the external application). Additionally, our transcriptome analysis demonstrated that auxin and jasmonic acid biosynthesis genes, as well as signaling transduction genes, were primarily activated in transgenic plants under salt stress, leading to activation of the cell wall biosynthesis or modification genes that promote plant growth. As a result, we investigated the mechanism through flavonoids enhance the salt tolerance, offering a theoretical foundation for enhancing salt tolerance in plants.

## Introduction

Various environmental stresses, such as strong winds, extreme temperatures, soil salinity, drought, and floods, have impacted agricultural crop production and cultivation. Soil salinity is one of the most devastating environmental stresses, causing significant reduction in cultivated land area, crop productivity, and crop quality (Shahbaz and Ashraf, 2013). High salinity is estimated to affect 20% of total cultivated and 33% of irrigated agricultural lands worldwide. Furthermore, the salinized areas are increasing at a rate of 10% annually due to various reasons, including low precipitation, high surface evaporation, native rocks weathering, poor irrigation systems and irrigation with saline water as well as due to poor agronomic practices (Shrivastava and Kumar, 2015). It has been estimated that more than 50% of the arable land would be salinized by the year 2050. Average yields for all important crops are only a fraction of record yields, ranging between 20% and 50% (Shrivastava and Kumar, 2015) and these losses are primarily due to drought and high soil salinity, environmental conditions that will worsen in many regions due to global climate change.

Salt stress has complex negative effects on many aspects of plant growth, including morphological, physiological, and biochemical processes (Akbarimoghaddam et al., 2011). In general, the two catastrophic primary effects of salt stress are physiological drought (osmotic stress) and ion toxic effects caused by ionic imbalance (Keisham et al., 2018), which could rapidly lead to cell death (Munns, 2002). Similarly, salt-tolerant plants have evolved various mechanisms to resist salt stress, such as the accumulation of small molecular osmoregulators to increase osmotic potential, a systematic ion transport system, and the separation of ion cellular compartments (Keisham et al., 2018). However, salt stress is always detrimental to plant growth, including ROS production (Das and Roychoudhury, 2014). Though a small amount of ROS can act as signaling molecules, coordinating an incredible range of diverse plant processes (Saed-Moucheshi et al., 2014), excessive ROS levels can cause cell death (Gill and Tuteja, 2010). Plants have evolved an elaborate enzymatic and non-enzymatic antioxidant system that, in conjunction with the ROS-producing enzymes, maintains ROS homeostasis in all cellular compartments to reduce the fatal damage caused by excessive ROS and to ensure the accurate execution of their signaling functions. SOD, catalase (CAT), ascorbate peroxidase (APX), and glutathione peroxidase (GPX) are the most common enzymatic systems (Apel and Hirt, 2004). Among the enzymatic systems, SOD can quickly convert O_2_•^-^ to H_2_O_2_, and the generated H_2_O_2_ is then converted to water and dioxygen by peroxidase and CAT (Gechev et al., 2006; Mittler, 2017). The non-enzymatic systems are mainly mediated by low molecular mass antioxidants, such as glutathione, ascorbic acid (AsA) and flavonoids, which are known to remove hydroxyl radicals and singlet oxygen (Gechev et al., 2006).

Flavonoids are a diverse group of plant secondary metabolites that are widely distributed throughout the plant kingdom. They are produced as a protective response to environmental stresses as a member of non-enzymatic systems. Flavonoid biosynthesis is upregulated under a wide range of abiotic stresses, such as ultraviolet (UV) radiation (Hectors et al., 2014) and slat stress (Colla et al., 2013). A previous study reported that NaCl and UV-B treatments increased the expression of genes involved in flavonol biosynthesis as well as flavonol content in *Reaumuria trigyna* (Zhang et al., 2017). Furthermore, flavonoids were significantly accumulated in leaves of several varieties of artichoke and cardoon treated exposed to salt stress (Colla et al., 2013). In addition, Chen et al. (2019) pointed that exogenous rutin supplementation effectively scavenged O_2_•^-^ and H_2_O_2_ and improved the salt tolerance of the rutin-reduced transgenic tobacco plants (Chen et al., 2019). All these previous studies have established and confirmed the role of flavonoids in salt tolerance.

Because of the abundance of flavonoids in *Apocynum venetum* leaves, the plant has been used in China to treat angiocardiopathies by regulating blood pressure, lowering blood fat, healing depression, and calming nerves (Xie et al., 2012). As a result, *A. venetum* has gradually evolved into an important plant used in traditional Chinese medicine with significant economic value. However, previous research found that *A. venetum* is not a typical halophyte because there is no suitable growth promoting salt concentration, and salt stress inhibited the normal growth and development of *A. venetum* seedlings and seed germination (Xu et al., 2020). Further investigation revealed that the leaf had the highest Na^+^ content in *A. venetum*, and the content of total flavonoids was negatively correlated with the content of sodium ions (Xu et al., 2021), confirming the importance of flavonoids in *A. venetum* salt tolerance. It was also confirmed in tobacco and *Arabidopsis* by overexpressing *AvF3H*, *AvF3*′*H*, *AvFLS*, and *AvF3GT*. Transgenic plants were more resistant to salt stress and contained more flavonoids than control plants (Wang et al., 2021; Xu et al., 2021). Though total flavonoid levels in *A. venetum* decreased under salt stress, some compounds, such as quercetin and kaempferol, increased significantly (Xu et al., 2020), and the enzyme synthase genes *AvF3’H*, *AvF3H*, and *AvFLS* showed multiple expression profiles (Xu et al., 2020; Xu et al., 2021). While we have preliminary data showing that these genes may improve tobacco and *Arabidopsis thaliana’s* ability to withstand salt stress under various salt stress concentrations and times, the precise mechanism by which they do so is still unknown. In this study, we systematically studied the expression profiling of major genes of flavonoid metabolic pathway in response to salt stress with the exposure time, and the mechanism of *AvF3’H*, *AvF3H*, and *AvFLS* enhancing plant salt tolerance was analyzed in *Arabidopsis thaliana*. This research highlights the roles of *AvF3’H*, *AvF3H*, and *AvFLS* in enhancing salinity stress tolerance, and provides a foundation for subsequent application.

## Materials and methods

### Plant materials

*Apocynum venetum* seeds collected from Dongying City in China’s Shandong Province and stored in the laboratory were used in this study. In transgenic experiments, *Arabidopsis thaliana* Columbia-0 (Col-0) was used as the wild type. Our previous study reported on these transgenic plants (*AvF3’H*-OE, *AvF3H*-OE, and *AvFLS*-OE) (Xu et al., 2021).

### Plant cultivation and salt stress treatment

*A. venetum* seeds were grown in vermiculite pots in a growth chamber at 24 °C with a 14-hour photoperiod and watered with 10% liquid MS. To investigate the expression patterns of *AvF3’H*, *AvF3H*, and *AvFLS*, six-week-old seedlings were watered with a 100 mM NaCl solution and the second completely spread (full grown) leaf from the top was collected at 0 min, 5 min, 15 min, 30 min, 1 h, 2 h, 4 h, 8 h, 12 h, 24 h, 48 h, and 72 h after watering with NaCl and immediately frozen in liquid nitrogen before storing them at -80 °C.

The sterile transgenic (*AvF3’H-OE, AvF3H-OE*, and *AvFLS-OE*) and wild type *Arabidopsis thaliana* seeds were grown in the growth chamber described above on 1/2 MS growth medium. After 10 days, some of these seedlings were transplanted into plastic trays. Three-week-old seedlings on plastic trays were evenly divided into two groups; one group was watered with water, while the other was watered with a 100 mM NaCl solution. After a week of treatment, the aerial phenotype was photographed with a digital camera (Canon 5D Mark III, Japan) and data was collected with Image J software. The seedlings were immediately frozen in liquid nitrogen and stored at -80 °C for further analysis, which included total flavonoid and compound composition content analysis, Na^+^ and K^+^ content detection, antioxidant enzymes activity test, ROS staining, and transcriptome analysis. The remainder of the original lot’s seedlings were transferred to 1/2 MS solid medium with or without 100 mM NaCl. Photographs of the seedlings were taken two weeks later, and data was collected using Image J software. Transgenic and WT seedlings were also transferred to 1/2 MS medium containing 100 mM NaCl with or without 100 µM rutin and eriodictyol for the flavone component complementation experiment. ROS staining was performed on both treated and untreated plants after a week. The flow chart of the experimental design is shown in **Supplementary File 1**.

### *Cis*-acting elements analysis of flavonoid synthetase enzyme genes promoter region

The *A. venetum* genome database (https://www.ncbi.nlm.nih.gov/genome/?term=txid377125[orgn]) was used to extract the 2000-bp sequences of flavonoid synthetase enzyme gene promoters from the NCBI database. The promoter *Cis*-acting elements were detected and identified using the PLACE database (http://bioinformatics.psb.ugent.be/webtools/plantcare/html/).

### Total flavonoid and main flavonoid compounds determination

The total flavonoids extraction method was based on a previous method (Zhang et al., 2015) with minor modifications. In brief, 50 mg dry sample powder was added to 3 mL 80% (w/v) methyl alcohol extracting in a water bath at 95 °C overnight. In a total of 110 µL supernatant, 440 µL NaNO_2_ (0.066 M, room temperature for 5 minutes), 60 µL AlCl_3_ (0.75 M, 6 minutes), and 400 µL NaOH (0.5 M) were added in turns. The absorbance of the mixture was measured at 510 nm. The total flavonoids were presented as mg rutin equivalents, i.e. mg/g DW. HPLC analysis method was used to detect the main flavonoid compounds content in transgenic *Arabidopsis* plants (Xu et al., 2020).

### Na^+^ and K^+^ content detection

The Na^+^ and K^+^ concentration measurement method was based on a previous method (Xu et al., 2020) with minor modifications. In brief, 0.25 g dried sample powder was digested in 5 mL of HNO_3_ at 110°C for about 6 hours until a colorless liquid was obtained. The cooled liquid mixture was diluted to 10 mL with deionized water. The Na^+^ and K^+^ content was then determined using an optical emission spectrometer (ICP, Optima 8000, PerkinElmer, USA)

### Antioxidant enzymes activity test

The SOD activity detection method was based on a previous method (Stewart and Bewley, 1980). SOD activity was defined as the ability of inhibiting the photochemical reduction of nitro blue tetrazolium. CAT activity was determined according to the method of (Patra et al., 1978), in which the ability of CAT was related to the extinction of H_2_O_2_. POD activity was measured by referring to the methodology of (Nickel and Cunningham, 1969).

### ROS staining and H_2_O_2_ and O_2_•^-^ content measurements

The histochemical staining methods using 3′3′-diaminobenzidine (DAB) and nitro blue tetrazolium (NBT) to detect H_2_O_2_ and O_2_•^-^, respectively, was referred (Zhao et al., 2016). Likewise, the H_2_O_2_ content detection method was based on a previous method reported by (Libik et al., 2005). The O_2_•^-^ content was detected with a specific kit (SA-2-G; Cominbio, Suzhou, China) following the manufacturer’s instructions.

### IAA and JA content detection

IAA and JA detection was done according to (Du et al., 2013) with miner modification. In brief, twelve seedlings of transgenic plants or WT samples under salt stress were ground into powder. 100 mg of powder were extracted twice with 900 µL of extraction buffer [methanol:H_2_O:acetonitrile = 90:9:1 (v/v)]. Quantification was performed in an ABI Qtrap6500 LC-MS system (Applied Biosystems, USA) with stable-isotope-labeled ABA and auxin as standards (Sigma).

### RNA extraction, qRT-PCR, and transcriptome analysis

Total RNA was extracted from samples with the EasyPure Plant RNA Kit (TransGen, ER301-01, Beijing, China) and genomic DNA contamination was removed using RNase-free DNase I (TransGen, K21109). First-strand cDNA synthesis and qRT-PCR amplification were performed as previously described by (Wang et al., 2018). **Supplementary File 2** lists the gene-specific primers that were used for qRT-PCR. The relative expression levels of target genes were calculated using the 2^-ΔΔCt^ method (Livak and Schmittgen, 2001) and normalized to those of the *AtTUBULIN* reference gene (Xu et al., 2017). For transcriptome analysis, the RNA purification, reverse transcription, library construction and sequencing were performed at Shanghai Majorbio Bio-pharm Biotechnology Co., Ltd. (Shanghai, China). Transcriptome sequencing data were analyzed on their online platform (www.majorbio.com) (Ren et al., 2022). The transcripts per million reads (TPM) was used to calculate the expression levels of each gene using RSEM (http://deweylab.biostat.wisc.edu/rsem/). Differential expression genes (DEGs) analysis was performed using DESeq2 (Love et al., 2014). The Q value ≤ 0.05 and |log2(Fold Change)| > 1 were used to identify DEGs. The GO and KEGG functional enrichment pathway analyses were performed using GO (https://github.com/tanghaibao/Goatools) and KOBAS (http://kobas.cbi.pku.edu.cn/home.do) tools.

### Data analysis

All charts (bar charts, boxplots, bubble charts, Venn diagrams and heatmap, so on) were drawn using Adobe Illustrator cs5 software and Majorbio Cloud Platform (www.majorbio.com). Significant differences among all the experimental treatments were determined using a one-way analysis of variance (ANOVA) followed by Tukey’s test at the probabilities of *p* < 0.05 level with SPSS version 17.0 software (SPSS, Chicago, IL, USA).

## Results

### Salt stress alters the expression patterns of *AvF3’H*, *AvF3H*, and *AvFLS*

**Figure 1:**
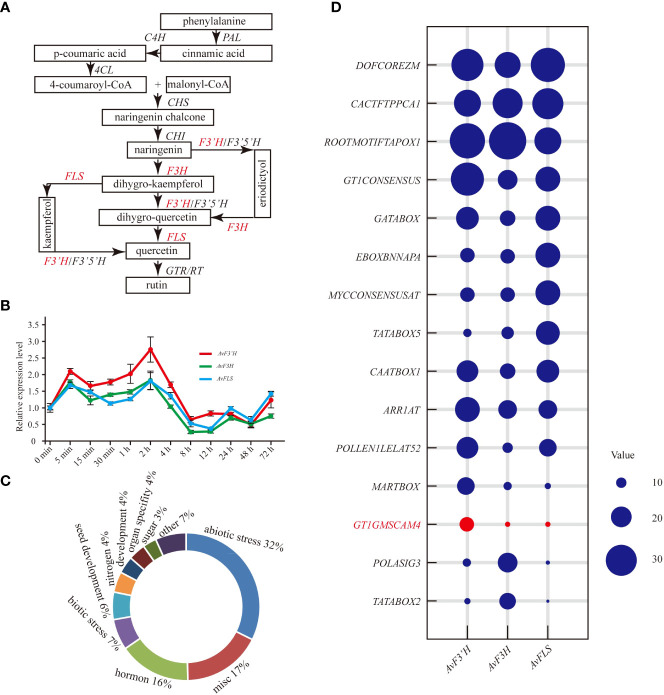
Expression patterns of *AvF3’H*, *AvF3H*, and *AvFLS* under salt stress. **(A)** Schematic of total flavonoid biosynthesis pathway (Koes et al., 2005; Yin et al., 2012) showed the catalytic action of *AvF3’H*, *AvF3H*, and *AvFLS*. **(B)** Expression levels of *AvF3’H*, *AvF3H*, and *AvFLS* in *A. venetum* leaves treated with 100 mM NaCl at different time. **(C)** Functional classification of *cis*-acting elements of *AvF3’H*, *AvF3H*, and *AvFLS* promoters. **(D)** Schematic illustration of the *cis*-acting elements distribution frequency of *AvF3’H*, *AvF3H*, and *AvFLS* promoters.

The main biosynthesis pathways of flavonoid metabolism are integrated in **Figure 1A**. The red marked genes (*AvF3’H*, *AvF3H*, and *AvFLS)* were the main focus in this study. The qRT-PCR results revealed that the expression patterns of these three genes in response to salt stress were similar. Under salt stress, they were induced to express at a high level for four hours. The expression level peaked at 5 minutes and 2 hours, with *AvF3’H* showing the highest level of expression compared to the other two. Salt stress inhibited the expression of three genes after four hours of exposure (**Figure 1B**). The expression patterns of other important genes in the flavonoid metabolic pathway were also studied, and the expression profiles were divided into two distinct groups. The expression patterns of *AvCHI*, *Av4CL3*, *AvF3’5’H*, *AvRT*, *AvCHS*, *AvPAL1*, and *AvC4H2* were similar to that of *AvFLS*, *AvF3H*, and *AvF3’H*, which showed a higher expression level in the first 4 hours, and reached the peak within 2 to 4 hours (**Supplementary File 3**). The expression patterns of others genes such as *Av4CL1*, *Av4CL2*, *AvC4H1*, *AvC4H3*, *AvPAL2*, *Av4CL4*, and *Av4CL* were clustered together. They showed rapid response to salt stress. The expression of these genes was rapidly induced in the first 30 minutes and then started to decline. It reached a low point at 4 hours, after which it gradually began to rise again (**Supplementary File 3**). The *Cis*-acting elements in the gene promoter, which usually respond to the types of binding transcription factors, were identified in the PLACE database to help elucidate the potential regulatory mechanism of these genes’ expression under salt stress. From the 2 kb promoters of AvF3’H, AvF3H, and AvFLS, a total of 133 different non-repetitive Cis-acting elements were identified (**Supplementary File 4**). These Cis-acting elements were found to be primarily involved in abiotic stress (32%), miscellaneous function (17%), hormones (16%), biotic stress (7%), and seed development (6%). (**Figure 1C**). The top ten Cis-acting elements in each gene were listed. “DOFCOREZM,” “CACTFTPPCA1,” and “ROOTMOTIFTAPOX1” were found in the highest frequency in all three genes, indicating that they may be involved in basic functions. The frequency of a salt-stress response element “GT1GMSCAM4” in AvF3’H, on the other hand, was significantly higher than in AvF3H and AvFLS (**Figure 1D**), suggesting that AvF3’H may play an important role in response to salt stress.

### Transgenic *Arabidopsis* plants exhibited better salt tolerance and had higher flavonoid levels

**Figure 2:**
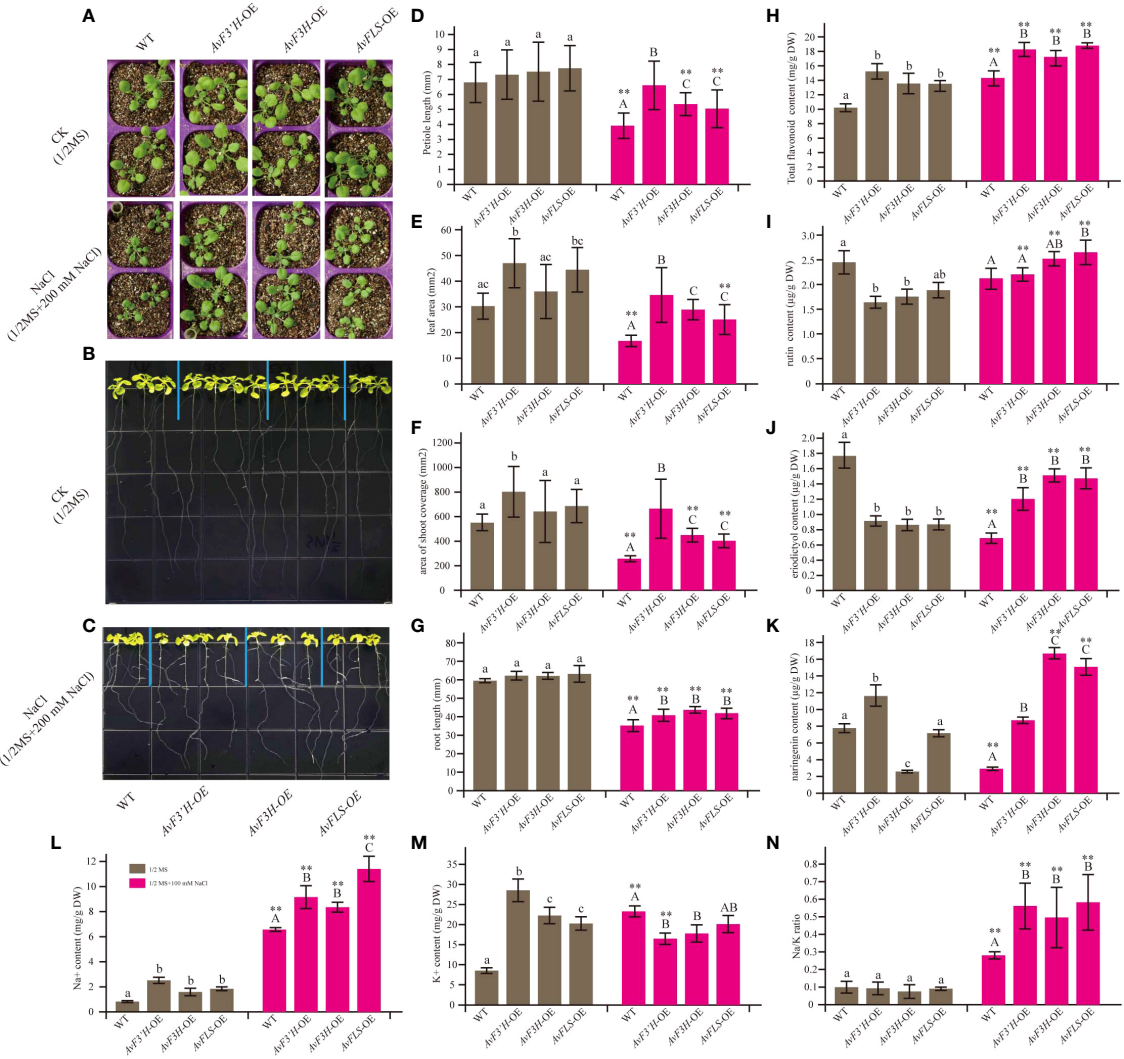
Overexpression of *AvF3’H*, *AvF3H*, and *AvFLS* increased the salt tolerance ability of *Arabidopsis*. **(A)** Seedlings aerial phenotype of transgenic plants and WT with and without salt stress. The root phenotype growing on normal 1/2 MS medium of transgenic plants and WT **(B)** and with 100 mM NaCl **(C)**. **(D)** Petiole length. **(E)** Leaf area. **(F)** The shoot coverage area. **(G)** Root length. **(H)** Total flavonoid contents. **(I)** Rutin contents. **(J)** Eriodictyol content. **(K)** Naringin content. **(L)** Na^+^ content. **(M)** K^+^ content. **(N)** Na/K ratio. Different lowercase letters indicated significant differences between transgenic plants and WT under normal condition (*P <* 0.05); different uppercase letters indicated significant differences between transgenic plants and WT under salt stress (*P <* 0.05), and ** indicated that there was a significant difference between the plants under normal condition and salt stress (*P <* 0.05).

OE-transgenic *Arabidopsis* plants were developed to investigate the potential roles of *AvF3’H, AvF3H*, and *AvFLS* in the response to salt stress. Semi-quantitative PCR was used to confirm the expression of *AvF3’H, AvF3H*, and *AvFLS* in transgenic plants (**Supplementary File 5**). Under normal growth conditions, the aerial parts of all three transgenic lines had longer petioles and larger leaves than WT, however, the statistical analysis results were not significant. Under salt stress, these parameters were significantly larger in transgenic plants than WT (**Figures 2A, D–F**), indicating that these three genes have a salt tolerance function. Phenotypic and statistical analysis revealed that there was no significant difference in the main root length of transgenic plants and WT under normal conditions (**Figures 2B, G**). Under 100 mM NaCl stress, however, transgenic plant roots were significantly longer than WT roots (**Figures 2C, G**). Furtherly, the endogenous flavonoid content results of these plants revealed that transgenic plants owed the higher flavonoid than WT both under normal and salt stress conditions (**Figure 2H**), suggesting a potential link between flavonoid content and salt tolerance. HPLC analysis was used to detect the flavonoids ingredients content. The content of rutin, eriodictyol, and naringerin in transgenic plants and WT were somewhat complex under normal conditions. However, all the transgenic plants showed significantly higher content compared to WT under salt stress (**Figures 2I–K**), suggesting the potential positive roles of them in response to salt stress.

Transgenic plants had significantly higher Na^+^ content under normal and salt stress than WT plants. Furthermore, the Na^+^ content of WT and transgenic plants was 3.6 to 7.9 times higher in saline than in normal conditions (**Figure 2L**). Interestingly, the K^+^ content in WT was 2.7 times higher under salt stress than under normal conditions, whereas the K^+^ content in transgenic plants decreased by 39.86%, 18.54%, and 33.46% in the three transgenic lines under salinity compared to normal conditions (**Figure 2M**), resulting in a higher Na/K ratio in transgenic plants under salt stress (**Figure 2N**). These findings suggested that overexpression of the flavonoid synthetase genes could increase the Na^+^ content and reduce the K^+^ content in transgenic plants, resulting in the ion homeostasis changes.

### Endogenous and exogenous flavonoids can reduce the ROS damage caused by salt stress

**Figure 3:**
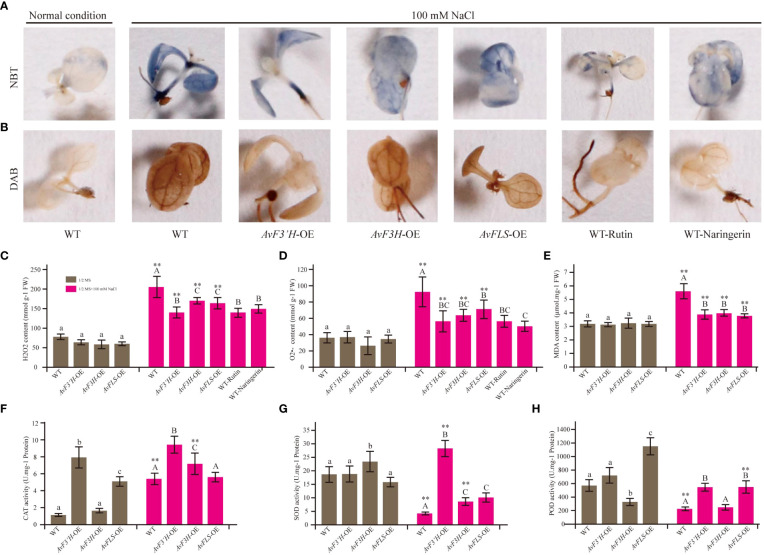
Transgenic plants and exogenous addition of rutin and naringerin alleviated ROS damage under salt stress. **(A)** NBT staining of transgenic plants and exogenous flavonoids application to plants under salt stress. **(B)** DAB staining. **(C)** H_2_O_2_ content. **(D)** O_2_•^-^ content. **(E)** MDA content. The CAT **(F)**, SOD **(G)** and POD **(H)** activity of transgenic plants and WT under normal and salt stress conditions. Different lowercase letters indicated significant differences between transgenic plants and WT under normal condition (*P <* 0.05); different uppercase letters indicated significant differences between transgenic plants and WT under salt stress (*P <* 0.05), and ** indicated that there was a significant difference between the plants under normal condition and salt stress (*P <* 0.05).

Salt stress is one of the detrimental environmental stresses inducing factors that lead to ROS accumulation. Thus, using 3,3’-diaminobenzidine (DAB) and nitrotetrazolium blue chloride (NBT) staining, histochemical analysis was used to investigate the effect of two ROS species, hydrogen peroxide (H_2_O_2_) and superoxide anion (O_2_•^-^). Under salt stress, the accumulation of brown and blue precipitates (showing DAB and NBT staining, respectively) in transgenic lines was much lower than in WT plants (**Figures 3A, B**). These findings suggest that transgenic plants’ improved salt stress tolerance may be due to reduced ROS production, which correlates with higher endogenous flavonoid levels in the three transgenic plants (**Figure 2H**). As expected, control plants with exogenous rutin and naringerin were significantly less damaged by ROS than those without under salt stress (**Figures 3A, B**). According to the spectrophotometry analysis results, the content of H_2_O_2_ and O_2_•^-^ that accumulated in the leaves of these transgenic plants showed no significant difference with that of WT under normal condition, however, significantly reduced compared with the WT plants under salt stress (**Figures 3C, D**). To evaluate the plants abiotic stress damage caused by salt stress, the MDA content was measured. As shown in **Figure 3E**, there was no significant difference in MDA content between the transgenic and WT plants under normal conditions. However, the MDA content in transgenic plants was significantly reduced (28.39% to 32.32%) compared to WT under salt stress. The antioxidant scavenging system, on the other hand, was activated in the transgenic plants. Under salt stress, the activity of CAT in both WT and transgenic plants was significantly higher than that under normal conditions, with the transgenic plants showing significantly higher activities than the control. Although the activities of SOD and POD were inhibited in WT and transgenic plants under salt stress (except SOD in *AvF3’H*-OE) compared to normal conditions, the activities of SOD and POD in transgenic plants were significantly higher than those in WT plants under salt stress (**Figures 3F, H**).

### Transcriptome analysis reveal the novel salt tolerance mechanism

**Figure 4:**
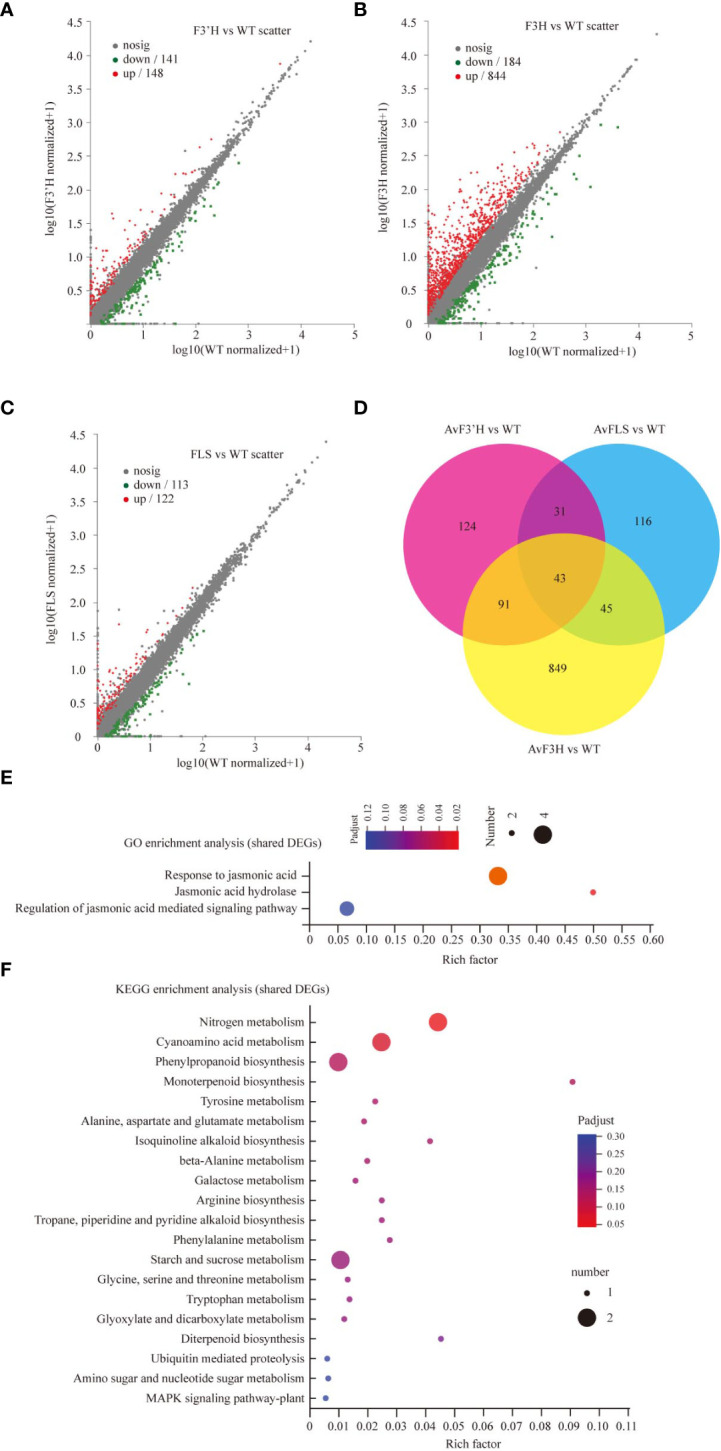
DEGs data annotation and enrichment analysis. The volcano plots showing the up- and down-regulated DEGs in comparisons *AvF3’H* vs WT **(A)**, *AvF3H* vs WT **(B)**, and *AvFLS* vs WT **(C)**. **(D)** Venn diagrams showing specific and shared DEGs in the 3 comparisons. GO **(E)** and KEGG **(F)** enrichment analysis of shared DEGs in the 3 comparisons (*p* < 0.05). The X-axis (Rich Factor) represents the percentage of DEGs belonging to the corresponding pathway.

In three independent experiments, we performed transcriptome deep sequencing (RNA-seq) analysis on transgenic and WT plants treated with 100 mM NaCl to further investigate the molecular mechanism of endogenous flavonoid accumulation in reducing the effect of salt stress. Three biological replicates of each treatment were compactly gathered together in the PCA diagram, indicating that the RNA-seq results were stable and reliable. Furthermore, while the four treatment samples were clearly separated, *AvF3H-OE* was separated from the other three samples, indicating that *AvF3H* overexpressing plants had a relatively distinct transcriptome profile from the other three samples (**Supplementary File 6**). We then identified differentially expressed genes (DEGs). A total of 1299 non-repeat genes were identified in transgenic plants when compared with WT (**Supplementary File 7**), of which 289 were identified in *AvF3’H* vs WT (148 up-regulated and 141 down-regulated) (**Figure 4A**), 1028 were identified in *AvF3H* vs WT (844 up-regulated and 184 down-regulated) (**Figure 4B**), 235 were identified in *AvFLS* vs WT (122 up-regulated and 113 down-regulated) (**Figure 4C**), suggesting that *AvF3H* overexpressing had a significant effect on gene regulatory networks. A venn map was used to identify shared DEGs among different comparison groups in order to investigate the general rule of flavonoid metabolism pathway related genes in response to salt stress. A total of 43 shared DEGs were identified, with 30 up-regulated and 13 down-regulated (**Figure 4D**, **Supplementary File 7**). GO enrichment analysis revealed that some JA related terms such as “Response to jasmonic acid”, “Jasmonic acid hydrolase”, and “Regulation of jasmonic acid mediated signaling pathway” were significantly enriched in these shared DEGs (**Figure 4E**). The most enriched KEGG pathway was “Nitrogen metabolism” pathway (**Figure 4F**). These findings suggested that JA and nitrogen metabolism play important roles in the response to salt stress. Furthermore, the top 10 enrichment GO and KEGG terms from each comparison group were integrated. Finally, a total of 23 non-repeated GO terms (**Supplementary File 8A**) and 18 KEGG pathways (**Supplementary File 8B**) were listed. We noticed that GO and KEGG terms of the *AvF3H* vs *WT* comparison group possessed more DEGs compared to the other two, which is consistent with the highest DEGs identified in this comparison group. GO terms “cell wall” and KEGG pathways “MAPK signaling pathway-plant”, “plant-pathogen interaction”, and “cyanoamino acid metabolism” were shared in all three comparison groups indicating the importance of these genes’ involvement in these terms. Furthermore, hormone related GO terms and KEGG pathways, such as “regulation of jasmonic acid mediated signaling pathway”, “jasmonic acid hydrolase”, “response to jasmonic acid”, “Plant hormone signal transduction”, and “alpha-Linolenic acid metabolism”, cell wall related terms such as “cell wall” and “plant-type cell wall”, flavonoid metabolism related terms such as “flavonoid biosynthesis”, “isoflavonoid biosynthesis”, and “phenylpropanoid biosynthesis” were significantly enriched in the transgenic vs WT groups, which suggests their involvement in salt tolerance. A total of 465 DEGs were involved in this enrichment GO or KEGG terms, and most of them were up-regulated in transgenic plants under salt stress compared to WT plants (**Supplementary File 8C**).

#### Na^+^ influx genes

The cell membrane acts as a barrier to material movement into and out of the cell. To investigate the cause of Na^+^ accumulation in transgenic plants, Na^+^ influx-related genes were investigated using transcriptome data. Despite the fact that no shared Na^+^ influx DEGs were identified, we discovered that a nonselective cation channel (NSCC) gene *GLR1.2* and an aquaporin gene *PIP2-3* were up-regulated in transgenic plants (**Table 1**), which corresponds to transgenic plants’ higher Na^+^ accumulation compared to WT plants (**Figure 2L**).

**Table 1:** Na^+^ influx and cell wall related DEGs.

Type	Gene ID	AvF3’H vs WT			AvF3H vs WT			AvFLS vs WT			Gene name
		Log2FC	Significant	Regulate	Log2FC	Significant	Regulate	Log2FC	Significant	Regulate	
Na+ influx	AT5G48400	0.8638	NO	UP	1.6713	YES	UP	1.3434	YES	UP	GLR1.2
	AT2G37180	1.3230	YES	UP	1.1256	YES	UP	0.8386	NO	UP	PIP2-3
Cell wall	AT5G05340	2.2444	YES	UP	1.0973	YES	UP	1.4533	YES	UP	PRX52
	AT4G38400	2.3441	YES	UP	3.4821	YES	UP	2.8022	YES	UP	EXLA2
	AT1G35140	2.4657	YES	UP	6.3258	YES	UP	0.9206	NO	UP	EXL1
	AT3G45970	1.4869	YES	UP	2.7161	YES	UP	0.7142	NO	UP	EXLA1
	AT4G25810	1.2934	YES	UP	1.8898	YES	UP	0.8822	NO	UP	XTH23
	AT1G76930	2.6961	YES	UP	0.2872	NO	UP	1.6286	YES	UP	EXT4
	AT4G30280	1.0969	YES	UP	2.2051	YES	UP	0.0653	NO	UP	XTH18
	AT5G57560	0.9355	NO	UP	4.4279	YES	UP	0.5555	NO	UP	XTH22
	AT4G30270	1.2495	YES	UP	2.1805	YES	UP	0.3895	NO	UP	XTH24
	AT1G32170	0.1994	NO	UP	1.8565	YES	UP	0.3401	NO	UP	XTH30

#### Endogenous flavonoids accumulation promotes cell wall expansion under salt stress

Among the shared DEGs, we discovered that two cell wall related DEGs *PRX52* and *EXLA2* were significantly up-regulated in transgenic plants under salt stress. In addition, eight cell wall expansin genes including three expansin-like genes (*EXL1*, *EXLA1* and *EXT4*), and five xyloglucan endotransglucosylase/hydrolase gene (*XTH18*, *XTH22*, *XTH23*, *XTH24*, and *XTH30*) were found to be significantly up-regulated in one or two comparison groups (**Table 1**).

#### Plant hormones improved salt tolerance

**Figure 5:**
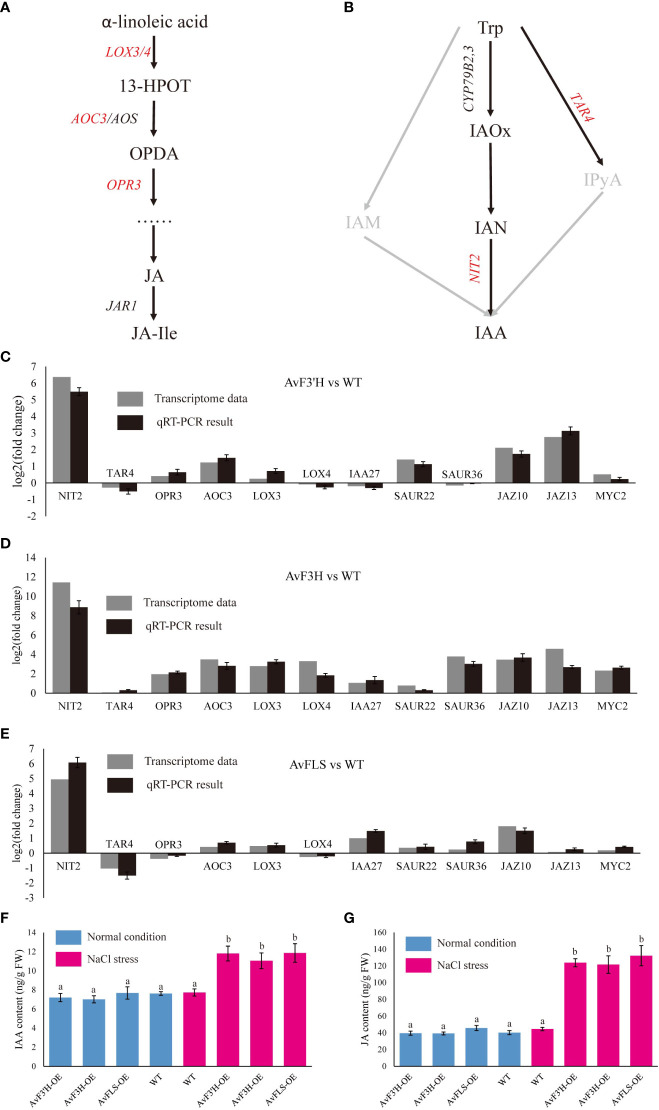
DEGs related to the plant hormone biosynthesis and signal transduction pathway. Diagram of jasmonic acid **(A)** and auxin **(B)** biosynthesis pathways. Validation of DEGs expression by qRT-PCR analysis of twelve DEG genes **(C–E)**. The IAA **(F)** and JA **(G)** content in transgenic plants and WT under normal growth and salt stress conditions. Different lowercase letters indicated significant differences between transgenic plants and WT (*P <* 0.05).

Jasmonic acid and IAA hormones are important plant growth regulators which also play a key role in plant response to abiotic stress. The synthesis and signaling transduction related genes of IAA and JA were screened from the transcriptome data in light of some IAA and JA related GO and KEGG terms enriched in transgenic plants compared to WT.As expected, *YUC*, *TRA*, and *TAA* genes in IAA synthesis pathway, and *LOX*, *AOS*, *AOC*, *OPR* and *DAD* genes in JA synthesis pathway were all identified. Although their expression levels tended to increase in transgenic plants (**Supplementary Files 9B, D**), only four (*LOX3*, *LOX4*, *AOC3*, and *OPR3*, **Figure 5A**) and two (*TAR2* and *NIT2*, **Figure 5B**) key synthesis genes among the nine and fourteen genes of JA and IAA synthesis related genes were identified as DEGs, respectively, and showed up-regulation in transgenic plants compared to WT (**Supplementary File 9A**, **Supplementary File 7**). A total of 13 IAA polarity distribution and transporter related genes such as *PIN*, *LAZY*, and *LAX* were identified, however, none of them were DEGs (**Supplementary File 9C**). Among 69 IAA signaling transduction related genes, only *SAUR22*, *SAUR36* and *IAA27* which were the early auxin-responsive genes showed significantly up-regulated in transgenic plants (**Supplementary File 9A**, **Supplementary File 7**). Three JA signaling transduction genes *JAZ10*, *JAZ13*, and *MYC2* were found to be significantly up-regulated in transgenic plants (**Supplementary File 9E**, **Supplementary File 7**). To validate the reliability of RNA-Seq data, the expression level of these twelve DEGs was examined by qRT-PCR. The similar expression trends of these DEGs were consistent with the Illumina sequencing (**Figures 5C–E**), indicating the dependability of the RNA-Seq data. The up-regulated of IAA and JA biosynthesis and signaling transduction pathway related genes indicated the accumulation of IAA and JA content in transgenic plants under salt stress. To confirm this hypothesis, IAA and JA levels were measured. Under normal growth condition, there was no significant difference in the amount of IAA and JA in transgenic plants and WT (**Figures 5F, G**). However, under salt stress, transgenic plants’ levels of IAA and JA significantly increased in comparison to WT, with an increase in IAA content of between 42.91% and 53.56% (**Figures 5F, G**). This was consistent with the up-regulated expression of IAA and JA synthetic pathway genes. On the other hand, under both normal and salt stress conditions, there was no significant different in the IAA and JA content of WT (**Figures 5F, G**). Accordingly, this indicates that the IAA and JA biosynthesis pathways were probably co-activated by the increase in flavonoid content in transgenic plants and salt stress.

### Role of flavonoids in salt tolerance

**Figure 6:**
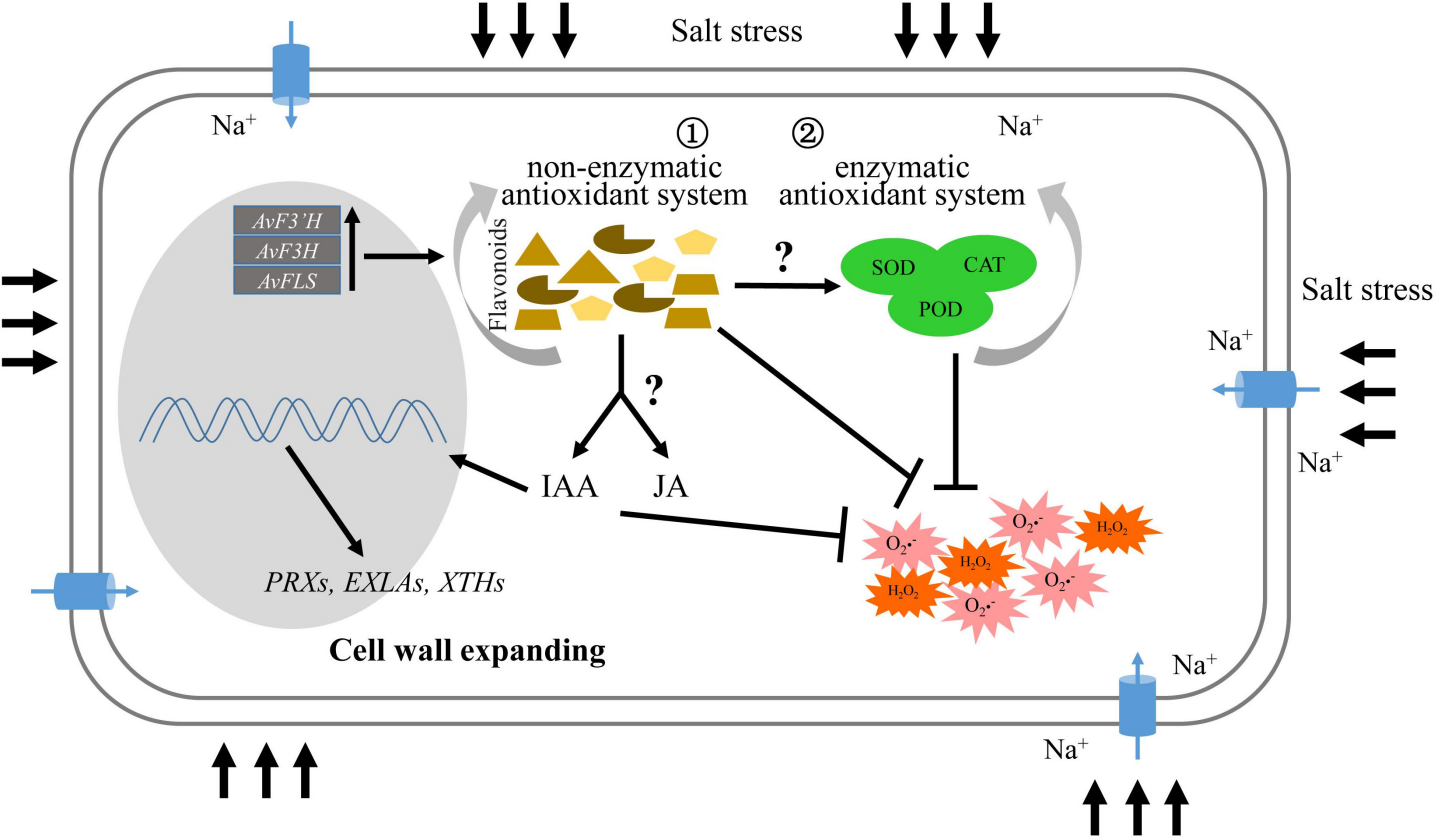
Schematic illustrating the role of flavonoids in salt tolerance.

Flavonoids, as an important member of the non-enzymatic antioxidant system, could directly eliminate the excess ROS produced by salt stress, working synergistically with the enzymatic antioxidant system. Importantly, flavonoids can also induce the expression of jasmonic acid and auxin synthesis genes *via* unknown pathways, resulting in the IAA and JA content increase, and activating the JA and IAA signal transduction pathways to promote the expression of cell wall biosynthesis or modification genes that promote cell wall expansion (**Figure 6**).

## Discussion

Flavonoids are found throughout the plant kingdom and are present in high concentrations in the epidermis of leaves and the skin of fruits. They are mainly classified into nine major subgroups: the chalcones, aurones, isoflavonoids, flavones, flavonols, flavandiols, anthocyanins, condensed tannins, and phlobaphene pigments (Winkelshirley, 2001). The biosynthesis of flavonoids is a very complex process that begins with the phenylpropanoid pathway. Chalcone synthase is the first committed enzyme in the biosynthesis of all flavonoids, producing a yellow-colored compound called chalcone. In most plants, chalcones are not the end-products, but the pathway proceeds with several enzymatic steps to other classes of flavonoids. The catalytic enzyme genes *F3H*, *FLS*, and *F3’H* are crucial for flavonoid branching. *F3H* catalyzes the formation of dihydroflavonol from flavanone. *F3’H* takes dihydrokaempferol or kaempferol as a substrate and converts it to dihydroquercetin and quercetin. *FLS* is the first dedicated enzyme for the biosynthesis of flavonols, branching from the main trunk route to the branch for anthocyanin formation (Pelletier et al., 1997). It could catalyze dihydroflavonols to form flavonols (Saito et al., 2013). Although all of the substrates are important secondary metabolites, the expression patterns of catalytic enzyme genes under salt stress were unclear. A 72-hour long expression experiment revealed multi expression level peaks at collecting time points for many flavonoid synthesis genes (**Supplementary File 3**), indicating the complex dynamic response of these genes to salt stress. Consistent with expression levels of these genes under salt stress, *AvF3’H* had a higher frequency of the salt-stress response element “GT1GMSCAM4” in the promoter region (**Figure 1D**). The cis-element “GT1GMSCAM4” was NaCl-induced and regulated by the GT-1-like transcription factor (Park et al., 2004). In general, salt stress would inhibit plant growth, resulting in shorter roots and stunted aerial parts. Our previous research found that 50 to 400 mM NaCl stress inhibited seed germination, plant height, root length, and leaf length of seedlings (Xu et al., 2020). The ion toxicity caused by the influx and accumulation of Na^+^ is a major cause of this phenomenon. Na^+^ influx across the plasma membrane in plants occurs primarily through nonselective cation channels (NSCCs) such as cyclic nucleotide-gated channels (CNGCs) and glutamate receptors (GLRs) (Keisham et al., 2018), as well as transporters such as HKTs and HAKs (Tester and Davenport, 2003; Deinlein et al., 2014). In addition, aquaporins have also been recently reported to be involved in Na^+^ uptake in plants (Byrt et al., 2017). Some of these Na^+^ influx genes were reported to be up-regulated in *A. venetum* under salt stress resulting in the Na^+^ excessive accumulation in leaves and dwarf plants caused by many cell wall biosynthesis or modification genes that were down-regulated (Xu et al., 2021). In this study, we also observed two potential Na^+^ influx genes *GLR1.2* and *PIP2-3* which were induced by salt stress in all the three transgenic lines (**Table 1**). Consistent with that, the Na^+^ contents in the transgenic plants were significantly higher than WT plants under salt stress (**Figure 2L**). Unlike our previous study, two types of cell wall modification genes (xyloglucan endotransglucosylase/hydrolases and cell wall expansin-related genes) were up-regulated in transgenic plants (**Table 1**). Plant cell walls provides mechanical strength to withstand the turgor pressure and determine the shape, size and function of cells. It is a complex dynamic structure and mainly composed by carbohydrate polymers such as cellulose, hemicellulose (mainly xyloglucan), and pectin, and structural proteins in variable amounts (Bellincampi et al., 2014). The xyloglucan endotransglucosylase/hydrolases (XTH) proteins have two distinct catalytic activities; xyloglucan endo-transglucosylase (XET activity) and xyloglucan endo-hydrolase (XEH activity). The XET domain cleaves a xyloglucan chain and rejoins the reducing end to another xyloglucan molecule, resulting in the elongation of xyloglucan. The XEH domain rejoins the xyloglucan reducing end to a water molecule resulting in irreversible xyloglucan chain shortening (Rose et al., 2002). Thus XTHs are considered key enzymes in the regulation of cell wall extensibility during cell growth (Cosgrove, 2005). Expansin related genes play a similar role (Marowa et al., 2020). The up-regulation of these cell wall modification related genes offered a reasonable explanation for the increased growth of transgenic plants under salt stress (**Figures 2A–G**).

The most significant difference between transgenic plants and other plants under salt stress is the high content of flavonoids. Flavonoids have been the subject of intense research interest because they were shown to have diverse functions in plants including oxidative damage protection, pathogen defense, interspecies communication, and even in auxin transport (Kuhn et al., 2011). Previous research suggested that flavonoids could influence auxin polar transport by competing for binding to auxin transport proteins with the auxin transport inhibitor 1-N-naphthylphthalamic acid (Murphy et al., 2002). Auxin transport is enhanced in the absence of flavonoids and decreased in the presence of excess flavonols (Peer et al., 2004). Furthermore, a previous study discovered that the accumulation of auxin in *rol1-2* seedlings, a flavonols aberrant accumulation mutant, was caused by a flavonol-induced modification of auxin transport (Kuhn et al., 2011). These studies confirmed the role of flavonoids in auxin transport. In this study, though no auxin transport related DEGs were identified, we found that the IAA synthase gene *NIT2* which was involved in an important IAA synthase branch hydrolysing indole-3-acetonitile (IAN) into IAA was up-regulated in transgenic plants (**Figures 5B–E**), which indicates that flavonoids are also involved in the regulation of auxin synthesis pathway. In fact, transgenic plants had higher levels of IAA than WT plants under salt stress (**Figure 5F**). Previous study have reported that when exogenous IAA was used in plants, the cell wall related genes that function on cellulose synthesis or modification, XyGs, pectins, structural proteins (EXPs), and peroxidases were up-regulated (Nemhauser et al., 2006). These results could explain why the expression of cell wall related genes was up-regulated in transgenic plant. However, the mechanism through which flavonoids promote the synthesis of IAA remains to be explored.

Jasmonic acid (JA) is an essential hormone involved in plant defense against herbivory and in responses to abiotic stress. Large-scale transcriptomic studies have shown that some JA-biosynthesis genes (e.g. *AOC1*, *AOC2*, *AOS*, *LOX3* and *OPR3*) are up-regulated in roots under salt stress (Kilian et al., 2007; Geng et al., 2013). In our study, *LOX3*, *AOC3*, and *OPR3* were also up-regulated in transgenic plants under salt stress (**Figure 5B**, **Supplementary File 7**). Some studies reported that exogenous JA could improve salt tolerance in many plants, which is consistent with the upregulation of JA synthetic genes. Maize seedlings treated with JA were found to significantly reduce the toxic effects of excess Na_2_CO_3_ on photosynthesis and plant growth parameters (Mir et al., 2018). In rice, post-application with exogenous JA can improve the leaf water potential, leaf photosynthetic rate, and maximum quantum yield of photosystem II (PSII) resulting in salt-stress alleviation, particularly in salt-sensitive cultivars (Kang et al., 2005). In *Arabidopsis*, overexpression of the wheat JA-biosynthesis gene *OPR1* reduces salt-mediated root growth inhibition (Dong et al., 2013). Furthermore, JA could effectively protect wheat seedlings from salt stress damage by increasing the activities of antioxidant enzymes and the concentration of antioxidative compounds to quench the excessive reactive oxygen species caused by salt stress, and this has practical implications for wheat cultivation in salt-affected soils (Qiu et al., 2014). These findings suggest that salt stress activates the JA biosynthesis and signaling pathway, causing physiological and growth changes in plants, which is consistent with this study.

Plant cells employ multiple mechanisms to regulate the levels of ROS to modulate signaling and prevent oxidative stress. Besides the antioxidant enzymes, synthesis of flavonoid metabolites that function as antioxidants *in vitro* is another important mechanism (Gayomba et al., 2017).Flavonoids are found in a variety of plant organs as well as different cells and cellular compartments, such as the cell wall, the vacuole of epidermal cells, and the external surface organs (Agati et al., 2012). Flavonoids occur as glycosides in healthy leaf cells, so the ROS scavenger capacity of flavonoids is dependent on the presence of the catechol group in the B-ring of the flavonoid skeleton(Rice-Evans et al., 1997). This also explained why the exogenous addition of rutin and naringerin reduced the ROS content of the control under salt stress (**Figure 3**).

## Conclusion

Flavonoids are important secondary metabolites of non-antioxidant enzyme system members that help to eliminate ROS generated by various abiotic stresses. Overexpression of flavonoids synthetase enzyme genes *AvF3’H, AvF3H*, and *AvFLS* increased not only total flavonoids content in transgenic plants, but also synergistically improved the antioxidant enzyme system, which improved salt tolerance. Similar to transgenic plants under salt stress, exogenous addition of flavonoids compounds like rutin and naringerin reduced ROS damage in plants. Furthermore, the biosynthesis and signaling transduction genes of IAA and JA was mainly up-regulated in transgenic plants under salt stress compared with WT, and the IAA and JA levels were significantly increased. Thus, the activated IAA and JA signaling pathway may activate cell wall biosynthesis or modification genes. As a result, the aerial part and root length of transgenic plants were greater than those of the control under salt stress.

## Data availability statement

The original contributions presented in the study are included in the article/**Supplementary Material**. The raw data of RNA-seq was uploaded into National Center for Biotechnology Information (NCBI) database with the BioProject accession number PRJNA945093 (https://www.ncbi.nlm.nih.gov/sra/PRJNA945093). Further inquiries can bedirected to the corresponding authors.

## Author contributions

ZX and LZ designed the study, TR, XL, JW, HY, CL, and CM carried out the study. MZ, ZX and PM wrote the manuscript. ZX and CM applied for funding. All authors contributed to the article and approved the submitted version.

## Supplementary material

Supplementary File 1 The graphical representation of experimental design.

Supplementary File 2 Primers used in this study.

Supplementary File 3 Heatmap of key genes expression in flavonoid synthesis pathway.

Supplementary File 4
*Cis*-acting elements of *AvF3’H*, *AvF3H* and *AvFLS* identified from the 2 kb length promoters.

Supplementary File 5 The expression level identification of *AvF3’H*, *AvF3H* and *AvFLS* in transgenic plants by semi-quantitative PCR.

Supplementary File 6 Principal component analysis (PCA) of the genes.

Supplementary File 7 Summary of transcriptome sequencing.

Supplementary File 8 The top 10 enrichment GO **(A)** and KEGG **(B)** terms of 3 comparison groups, respectively, and heatmap analysis of enrichment DEGs **(C)**.

Supplementary File 9 Expression level heatmap of genes related the biosynthesis, transport, and signaling transduction of IAA and JA.
