# Supplementary figures and images for: Heterologous overexpression of Apocynum venetum flavonoids synthetase genes improves Arabidopsis thaliana salt tolerance by activating the IAA and JA biosynthesis pathways
Source: Front Plant Sci. 2023 Mar 27;14:1123856. doi: 10.3389/fpls.2023.1123856 (PMC10083295; doi:10.3389/fpls.2023.1123856)

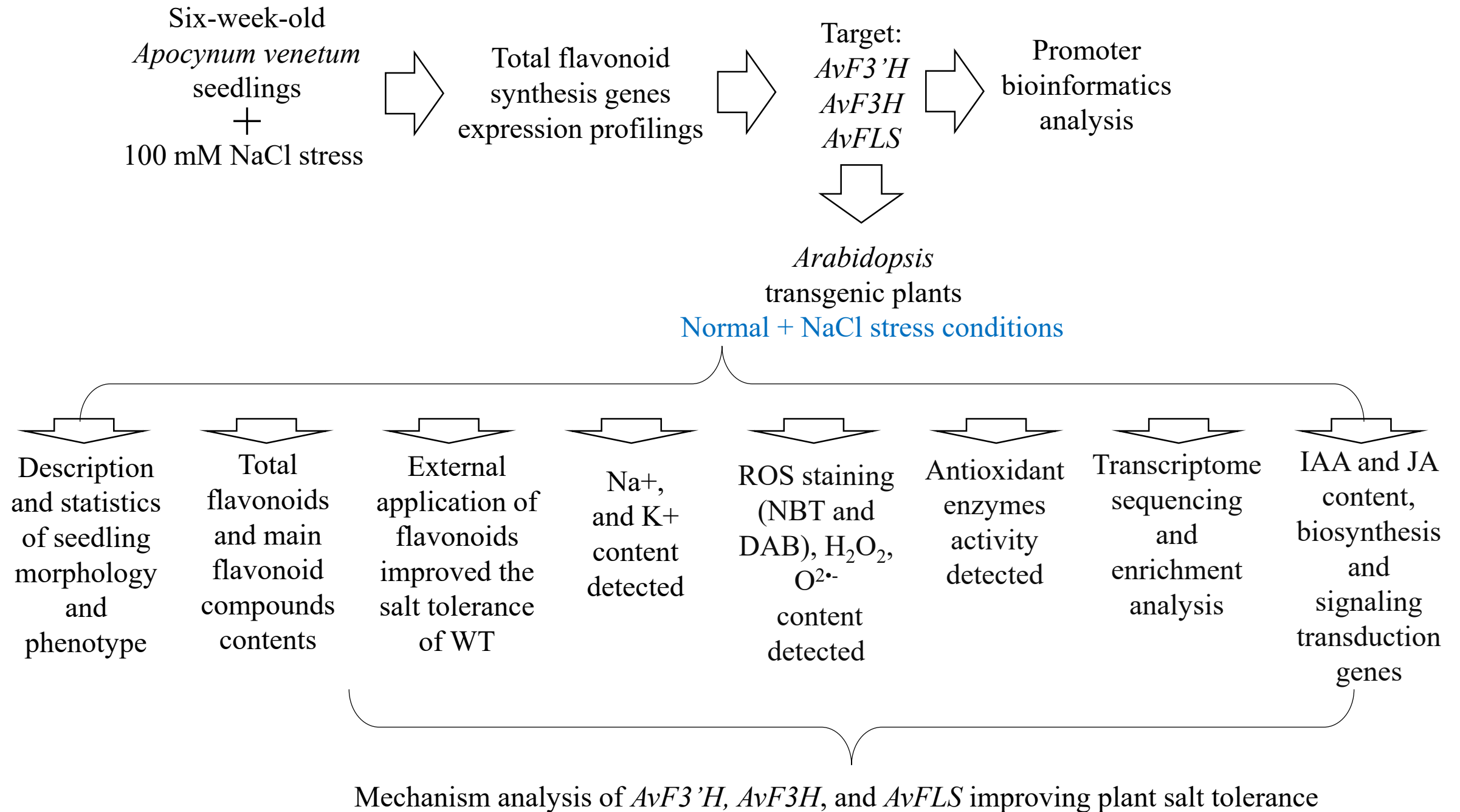

Supplement: Supplementary File 1 — The graphical representation of experimental design. [file DataSheet_1.pdf]

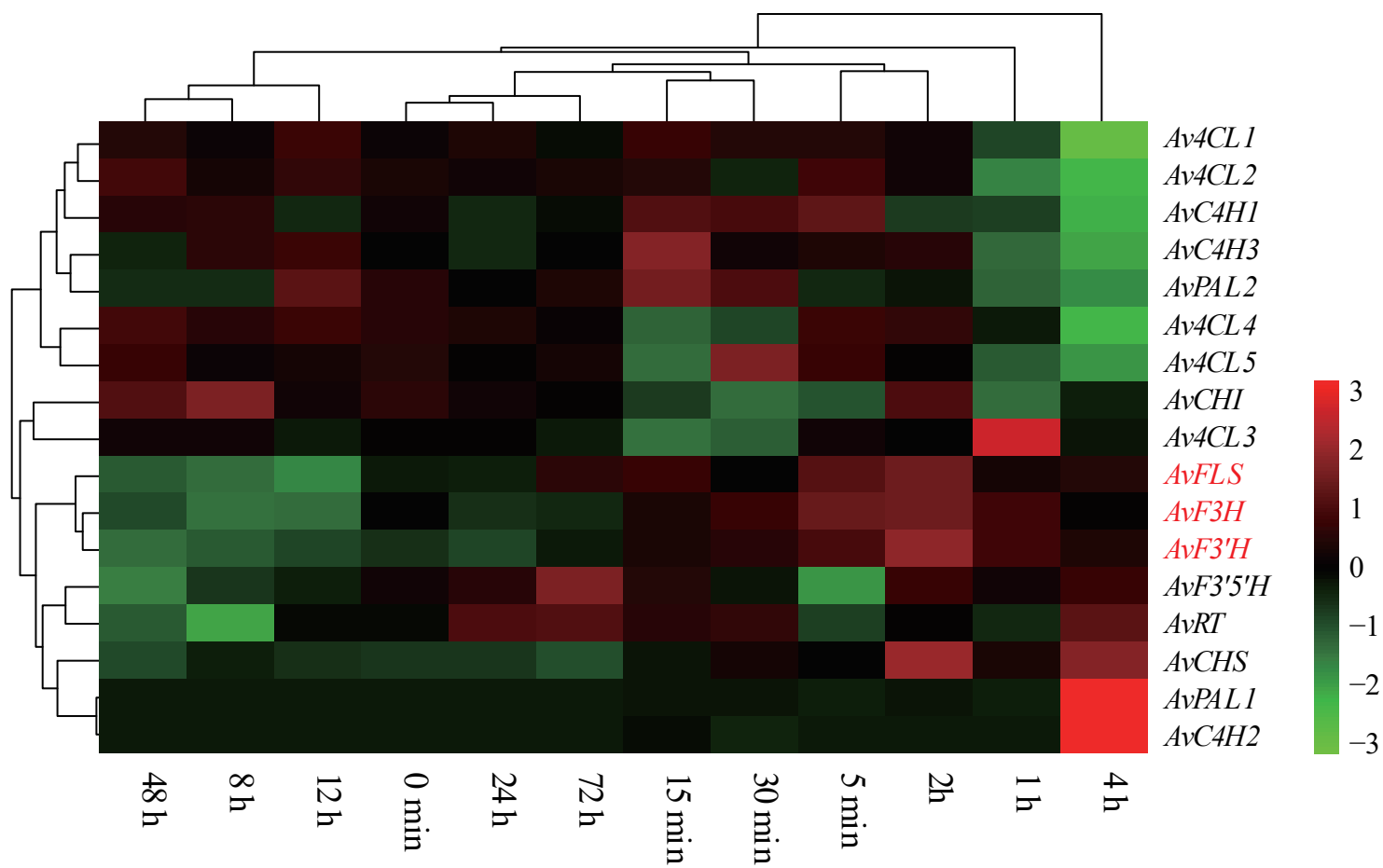

Supplement: Supplementary File 2 — Primers used in this study. [file DataSheet_2.pdf]

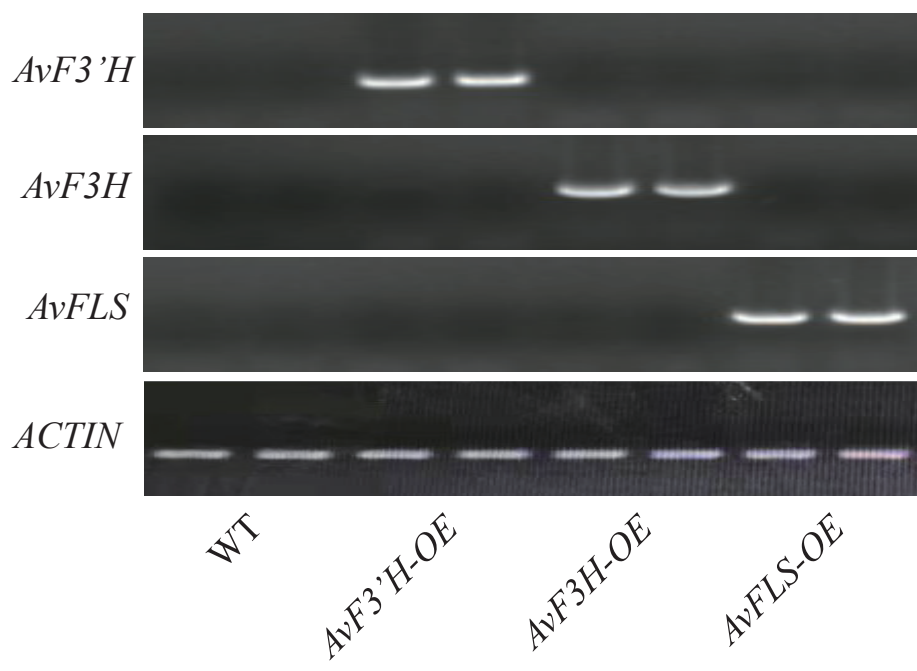

Supplement: Supplementary File 3 — Heatmap of key genes expression in flavonoid synthesis pathway. [file DataSheet_3.pdf]

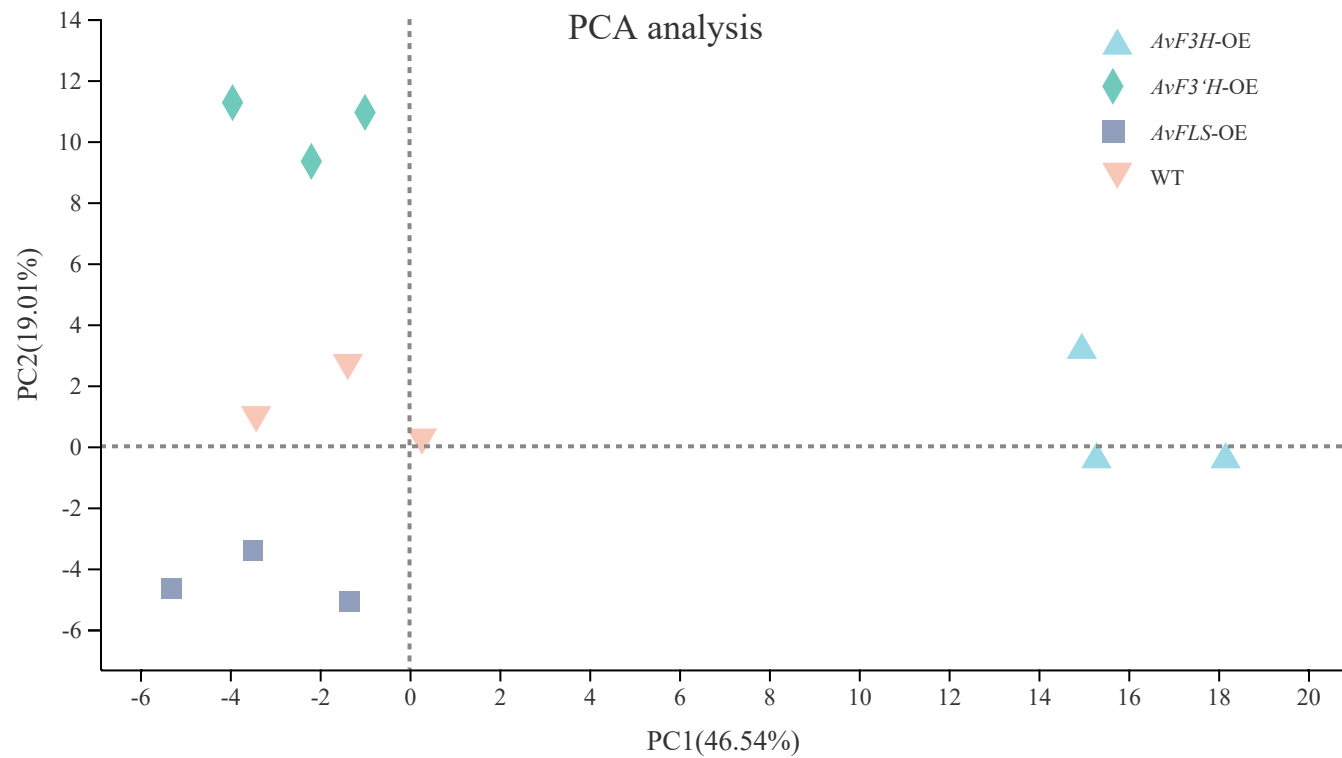

Supplement: Supplementary File 4 — Cis-acting elements of AvF3’H, AvF3H and AvFLS identified from the 2 kb length promoters. [file DataSheet_4.pdf]

A

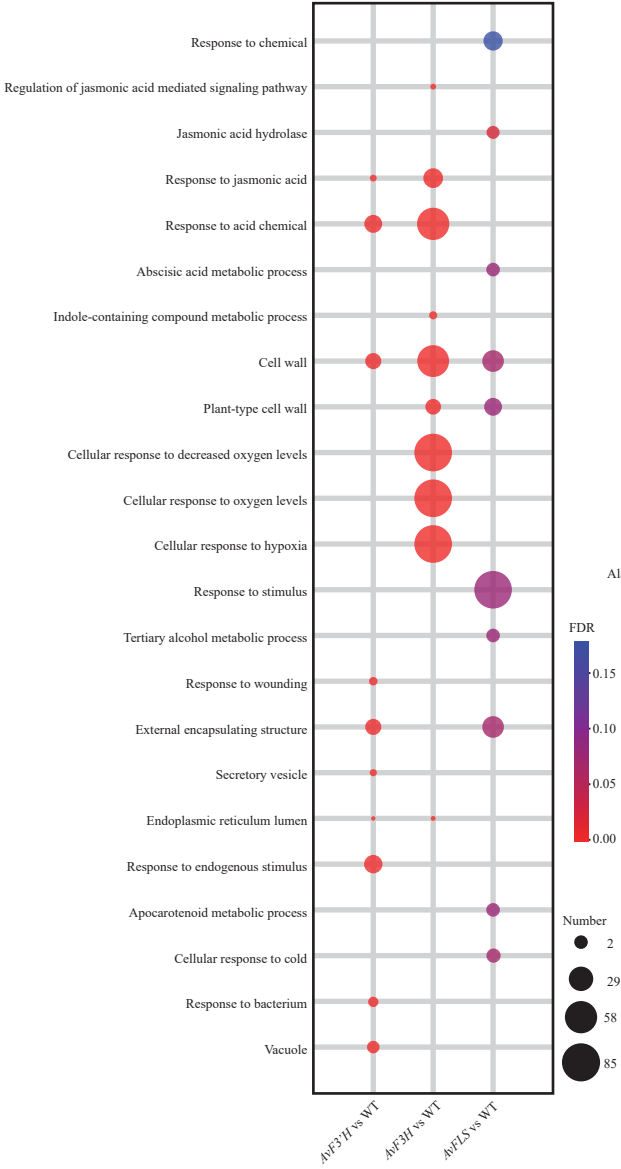

B

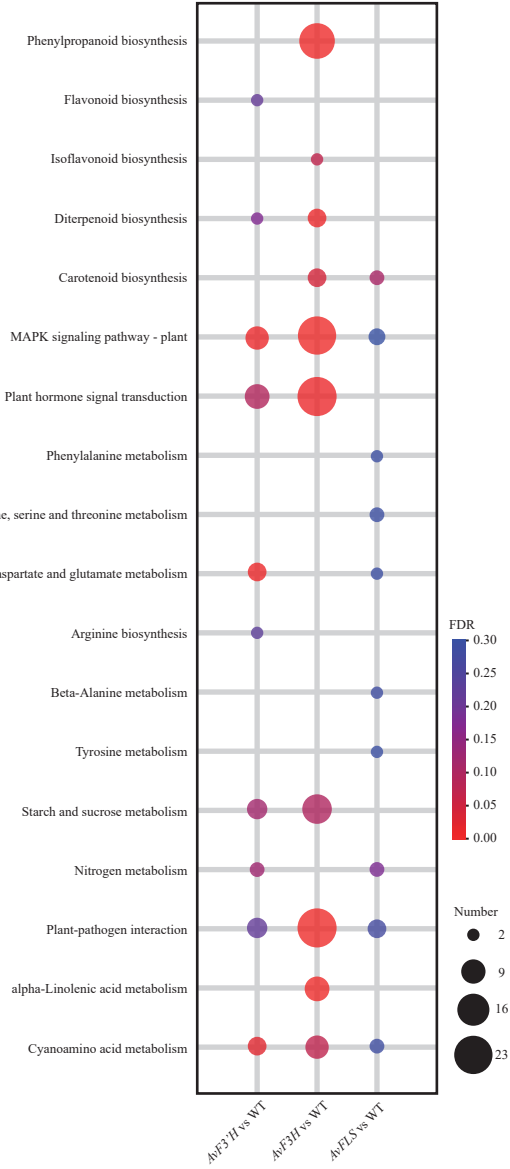

C

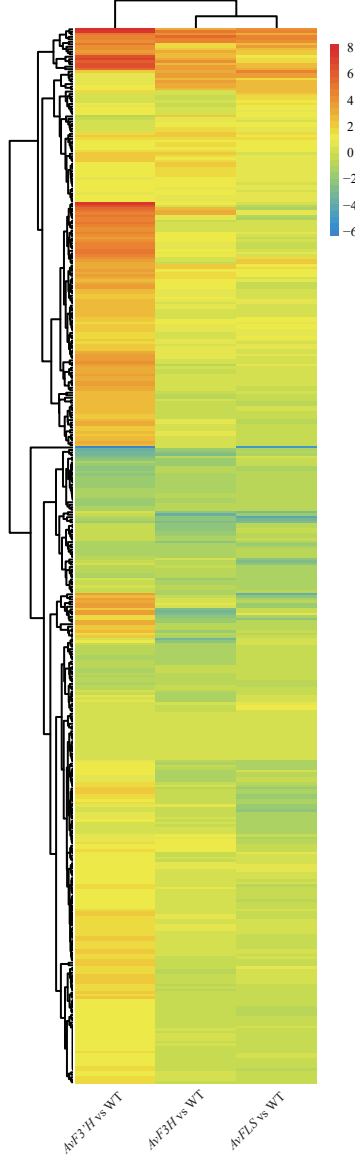

Supplement: Supplementary File 5 — The expression level identification of AvF3’H, AvF3H and AvFLS in transgenic plants by semi-quantitative PCR. [file DataSheet_5.pdf]

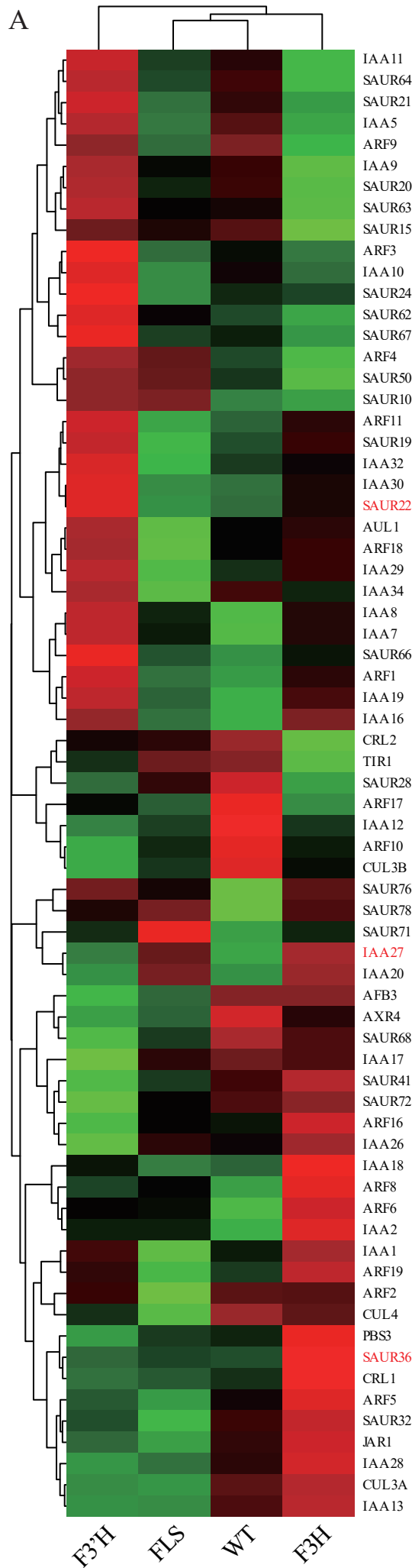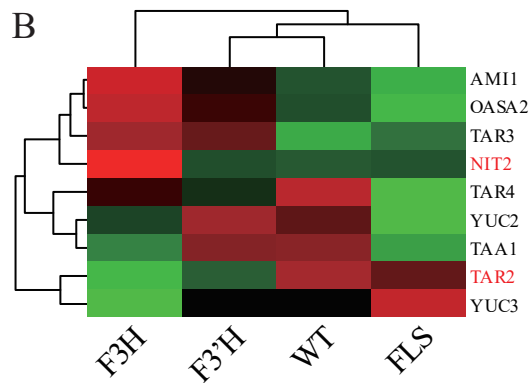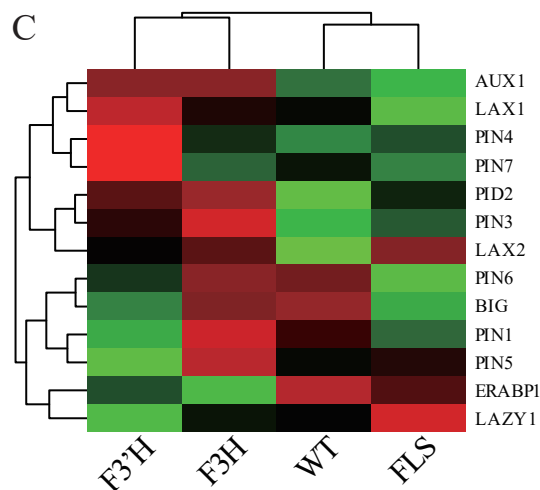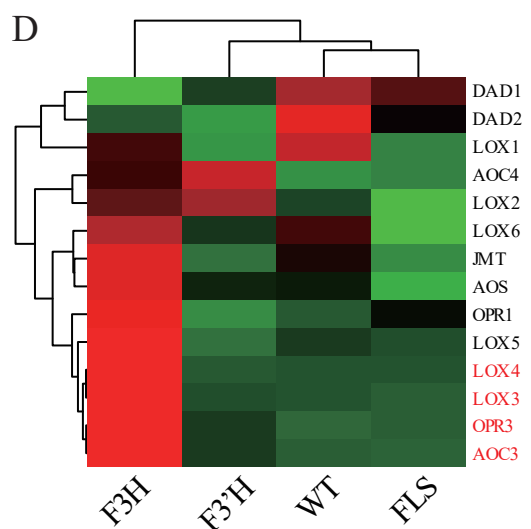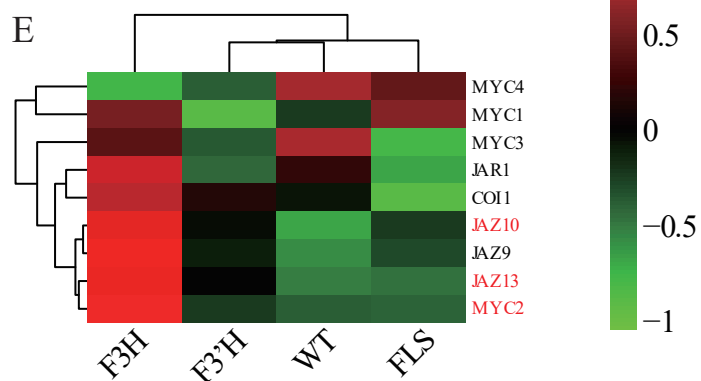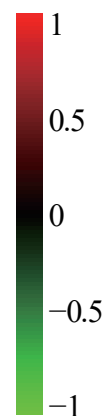

Supplement: Supplementary File 6 — Principal component analysis (PCA) of the genes. [file DataSheet_6.pdf]
